# Feasibility of fluorescence imaging at microdosing using a hybrid PSMA tracer during robot-assisted radical prostatectomy in a large animal model

**DOI:** 10.1186/s13550-022-00886-y

**Published:** 2022-03-07

**Authors:** Paolo Dell’Oglio, Danny M. van Willigen, Matthias N. van Oosterom, Kevin Bauwens, Fabian Hensbergen, Mick M. Welling, Huijbert van der Stadt, Elise Bekers, Martin Pool, Pim van Leeuwen, Tobias Maurer, Fijs W. B. van Leeuwen, Tessa Buckle

**Affiliations:** 1 Interventional Molecular Imaging Laboratory, Department of Radiology, Leiden University Medical Center, Albinusdreef 2, 2300 RC Leiden, The Netherlands; 2 Department of Urology, ASST Grande Ospedale Metropolitano Niguarda, Milan, Italy; 3 Department of Urology, Netherlands Cancer Institute - Antoni van Leeuwenhoek Hospital, Amsterdam, The Netherlands; 4 ORSI Academy, Melle, Belgium; 5 Design and Prototyping, Leiden University Medical Center, Leiden, The Netherlands; 6 Department of Pathology, Netherlands Cancer Institute - Antoni van Leeuwenhoek Hospital, Amsterdam, The Netherlands; 7 Department of Clinical Pharmacy and Toxicology, Leiden University Medical Center, Leiden, The Netherlands; 8 Martini-Klinik, Universit¨Atsklinikum Hamburg-Eppendorf, Hamburg, Germany

**Keywords:** Prostate cancer, PSMA, Image-guided surgery, Fluorescence imaging, Microdosing, Robot-assisted surgery

## Abstract

**Background:**

With the rise of prostate-specific membrane antigen (PSMA) radioguided surgery, which is performed using a microdosing regime, demand for visual target confirmation via fluorescence guidance is growing. While proven very effective for radiotracers, microdosing approaches the detection limit for fluorescence imaging. Thus, utility will be highly dependent on the tracer performance, the sensitivity of the fluorescence camera used, and the degree of background signal. Using a porcine model the ability to perform robot-assisted radical prostatectomy under fluorescence guidance using the bimodal or rather hybrid PSMA tracer (^99m^Tc-EuK-(SO_3_)Cy5-mas_3_) was studied, while employing the tracer in a microdosing regime. This was followed by ex vivo evaluation in surgical specimens obtained from prostate cancer patients.

**Results:**

T_50% blood_ and T_50% urine_ were reached at 85 min and 390 min, in, respectively, blood and urine. Surgical fluorescence imaging allowed visualization of the prostate gland based on the basal PSMA-expression in porcine prostate. Together, in vivo visualization of the prostate and urinary excretion suggests at least an interval of > 7 h between tracer administration and surgery. Confocal microscopy of excised tissues confirmed tracer uptake in kidney and prostate, which was confirmed with PSMA IHC. No fluorescence was detected in other excised tissues. Tumor identification based on ex vivo fluorescence imaging of human prostate cancer specimens correlated with PSMA IHC.

**Conclusion:**

Intraoperative PSMA-mediated fluorescence imaging with a microdosing approach was shown to be feasible. Furthermore, EuK‐(SO_3_)Cy5‐mas_3_ allowed tumor identification in human prostate samples, underlining the translational potential of this novel tracer.

*Trial registration* Approval for use of biological material for research purposes was provided by the Translational Research Board of the Netherlands Cancer Institute-Antoni van Leeuwenhoek hospital (NKI-AvL) under reference IRBm19-273 (22/10/2019).

## Background

With the introduction of imaging agents based on prostate-specific membrane antigen (PSMA) inhibitors, PSMA-mediated surgery is starting to claim a prominent role in prostate cancer (PCa) treatment [1, 2]. Several radioactive PSMA tracers have been applied in patients with radioguidance in mind [3], for example ^111^In-PSMA I&T [4], ^111^In-PSMA-617 [5], ^99m^Tc-PSMA I&S [6], and ^68^Ga-PSMA-11 [7], all of which are applied within the microdosing regimen (≤ 100 µg, or ≤ 30 nmol for protein-based therapies/patient; [8, 9]). This type of dosing is considered favorable with regard to legislation and cost of toxicity studies (e.g., reduced chance of side effects). ^99m^Tc-PSMA I&S, in particular, has proven great potential in large patient groups for salvage nodal resection [10] and salvage surgery within the seminal vesicle bed [11, 12]. In parallel, developments in medical devices [13] have made it possible to extend PSMA-targeted radioguided surgery from open to minimally invasive procedures such as robot-assisted radical prostatectomy (RARP) [14].

To facilitate real-time optical guidance during surgery without false negative outcome, various fluorescent derivatives of the above-mentioned small-molecule PSMA-inhibitors have been reported in the literature, as summarized by Hensbergen et al. [15]. This review also underscores that fluorescent agents based on PSMA inhibitors or antibodies are commonly used at concentrations that reach therapeutic levels ([15, 16]; 2.5 mg/kg in patients). These high doses also tend to go hand in hand with, i.e., high kidney retention and renal excretion, and may even exert unwanted pharmacological activity [6, 15, 17]. When realizing that the ureter is standardly dissected during RARP, spillage of fluorescence-containing urine into the surgical field can cause false positive findings [18]. This feature is also highly reliant on the biological half-life of a tracer. Since higher molar activities help reduce receptor saturation and thus improve the diagnostic potential of PSMA-radiotracers [19], one can wonder if administration of > 100 µg of a PSMA-tracer would not automatically lead to overdosing and increase background signals. In addition, clinical studies indicate that microdosing should be feasible for fluorescent agents [20, 21].

Since the successes obtained in sentinel node biopsy studies, there is an ongoing trend in image-guided surgery to move away from individual modalities and use hybrid techniques that integrate fluorescence and radionuclide detection [22]. This not only offers practical advantages with regard to image guidance, but it can also be used to perform quantitative biodistribution studies [15] and to connect the dosing regimens. A number of small molecule hybrid PSMA tracers have been reported in preclinical studies, two of which have made it to early clinical studies [15, 23, 24]. Especially in the latter, intraoperative fluorescence imaging seems to pose the biggest challenge for use in fluorescence-guided resections.

Recently, we reported the design of the hybrid PSMA tracer ^99m^Tc-EuK-(SO_3_)Cy5-mas_3_ [25]. Given the validated performance in mouse tumor models (of human origin) and the above-mentioned sensitivity challenges for fluorescence imaging, one of the translational challenges was to assess if this tracer allows for accurate fluorescence guidance at a microdosing regimen. With the difference between 25 g mice and > 60 kg man in mind, these evaluations were performed in a 35–40 kg porcine model. Fluorescence imaging was performed using a prototype clinical grade fluorescence laparoscope and custom image analysis software. To obtain an early compatibility with human disease [26], ex vivo incubation of clinical PCa samples was performed as a pilot/indication of feasibility.

## Materials and methods

### Tracer synthesis

EuK‐(SO_3_)Cy5‐mas3 (MW = 1411.6 g/mol; *K*_D_ = 19.2 ± 5.8 nM) was synthesized according to previously described methods [25].

### Imaging hardware

For imaging of white light and Cy5 a clinical grade prototype IMAGE 1 S camera system equipped with a 0° laparoscope was used (KARL STORZ) that was designated for preclinical or clinical use. For Cy5 imaging this setup was complemented with a Cy5-specific click-on filter (Cat. No. 20100034 [26]) that had to be removed to allow for white light imaging, or an in-house developed filter wheel that could manually be switched between white light or Cy5 mode. Both filter options were placed in between the camera and the laparoscope.

### Imaging software

In-house developed image processing software was used to create color-coded heat-map and real-time representation of the signal-to-background ratio (SBR) based on the intensity of the fluorescence signal [27]. Differences in fluorescence signal intensity were represented in an intensity-based scale-bar in real time. A pseudo-colored fluorescence overlay allowed real-time visualization of the distribution of the fluorescence signal within the tissue sample.

Image processing software was written in C++-programming language using open-source computer vision libraries (OpenCV).

### Porcine model

Overall, 15 non-tumor-bearing pigs were used. Animals were bred and kept in accordance with Belgium law in and by a licensed establishment for use of experimental animals. Pigs were housed at the animal facility at ORSI Academy (Melle, Belgium) until used for surgical training and imaging experiments (35–40 kg per animal)**.** Imaging experiments were performed after completion of surgical training (duration training: 4–7 h). All animals remained under anesthesia for the entire duration of the experiment and were euthanized when the examination was completed. Given the similarity in physiology and metabolism, blood, and urine sampling of both female (*N* = 10) and male animals (*N* = 5) were used for assessment of tracer kinetics, the male animals were used to evaluate in vivo fluorescence imaging of, i.e., basal PSMA expression levels in the prostate [28–30].

Experiments were approved by the local ethics committee of Gent University (EC2019/79) and were performed in accordance with the Experiments on Animals Act (Wod, 2014), the applicable legislation in Belgium and in accordance with the European guidelines (EU directive no. 2010/63/EU) regarding the protection of animals used for scientific purposes.

### Tracer administration and blood/urine sampling

100 µg EuK‐(SO_3_)Cy5‐mas_3_ [25] was dissolved in 1.5 mL 0.9% saline solution and administered intravenously. Blood samples were obtained from the ear at set time intervals between *T* = 0 (prior to tracer administration) and *T* = 360 min after tracer administration. Blood samples were obtained using a 1-mL syringe with a 21G needle and then transferred into BD Vacutainer K2E tubes (Franklin Lakes, New Jersey, USA). Samples were shaken vigorously to activate anti-clotting and subsequently stored at 4 °C.

To monitor the animals well-being, breathing and heart rate were monitored while under anesthesia. Urine samples were collected via a catheter between *T* = 0 and *T* = 420 min after tracer administration. Restrictions in facility access and timeframe of anesthetics prohibited evaluation of urine levels of EuK‐(SO_3_)Cy5‐mas_3_ beyond this time point. In female animals the catheter was placed in the urethra. As the anatomy of male pigs prevents placement of a catheter in the same way, a needle connected to tubing was inserted into the abdomen via the assistant 12-mm robotic surgical portal, which was then inserted directly into the bladder. Urine samples were collected using 2-mL syringes and transferred into plastic containers before being stored at 4 °C.

### Quantification of tracer levels in porcine blood and urine

Following centrifuging of the blood samples at 16,100 RCF for 15 min, 100 µL of the plasma was transferred to a well of a white 96-wells Lumitrac plate (Greiner). 300 µL of each urine sample was transferred to a white 96-wells Lumitrac plate. The fluorescence emission at 664 nm (excitation at 648 nm) was then quantified using a LS-55 fluorescence spectrometer (PerkinElmer). The time point at which 50% of the maximum fluorescence signal was cleared from the blood and urine (T_50%blood_ and T_50%urine_) was determined by fitting a sigmoid function on the descending data points in MATLAB software.

### Surgery and in vivo imaging

In vivo imaging of the prostate was performed during RARP in five male pigs, after obtaining the last urinary sample, using a da Vinci Si system (Intuitive Surgical Inc.). White light and Cy5 far-red fluorescence imaging were performed at 4–7 h after tracer administration using a clinical-grade IMAGE 1 S camera system equipped with a 0° laparoscope (KARL STORZ), as specified above. Animals were maintained under isoflurane anesthesia for the complete duration of the surgery and were euthanized before awakening.

### Ex vivo and fluorescence imaging of porcine and human PCa tissue

Following RARP, tracer uptake in excised tissues (prostate, kidney, ureter, liver, abdominal fat, muscle, splenic tissue, and salivary gland) was evaluated ex vivo using a clinical grade Cy5 prototype laparoscopic system. Samples were imaged immediately after excision and then stored at − 20 °C. Prior to fluorescence confocal imaging samples were thawed, cut into thin slices, and placed on a 35-mm culture dish that contained a glass insert (MatTek co). Fluorescence confocal images were acquired using a Leica SP8 WL microscope (Leica Microsystems) at 10× or 63× magnification (*λ*_ex_ 633 nm, *λ*_em_ 650–700 nm). Images were analyzed using Leica Confocal Software (Leica Microsystems).

Human prostate samples containing tumor tissue (*N* = 3) were obtained from prostate cancer patients after undergoing RARP. Approval for use of biological material for research purposes was provided by the Translational Research Board of the Netherlands Cancer Institute-Antoni van Leeuwenhoek hospital (NKI-AvL) under reference IRBm19-273. Patient approval for (ex vivo) use of tissue specimens was (digitally) acquired at the admittance desk of the NKI-AvL. All specimens were cut in half at the pathology department before being incubated in a solution containing 500 nM (20 mL) of EuK‐(SO_3_)Cy5‐mas_3_ for 15 min and rinsed twice with PBS to remove unbound tracer. Prostate samples were then imaged (dissection plane, whole prostate) using the clinical Cy5 system. Hereafter, samples were formalin-fixed and then paraffin-embedded. Real-time image processing of the fluorescence image was applied to provide a representation of the SBR and fluorescence signal intensity differences within the tissue [27].

### Immunohistochemistry

For evaluation of PSMA expression in porcine tissues 4-µm sections were cut. Antigen retrieval was performed using 0.01 M sodium citrate buffer, pH 6.0. Samples were then rinsed with 1 × TBS with Tween (TBST). Samples were incubated with the primary antibody (recombinant murine anti-human IgG1 FOLH1/PSMA, epitope aa44-750, Clone GCP-04, LifeSpan Biosciences) for 45 min, washed with 1X TBST and then incubated with a biotinylated secondary antibody (goat anti-mouse/rabbit IgG HRP, BrightVision, Immunologic). Staining with an Ab non-specific for pig PSMA (clone 3E6, Agilent/DAKO) was used as negative control. After rinsing with TBST, alkaline phosphatase streptavidin was applied and incubated for 30 min at room temperature. Slides were rinsed again and then incubated with alkaline phosphatase chromogen substrate for 30 min and washed with distilled water. Samples were covered with a coverslip and imaged on a slide scanner (Pannoramic® 250 Flash III reader, 3DHISTECH).

Histopathological examination of human prostate samples was performed as per standard of care by an experienced uropathologist. Immunohistochemistry of prostate samples was performed on a BenchMark Ultra autostainer (Ventana Medical Systems). In brief, paraffin sections were cut at 3 µm, heated at 75 °C for 28 min and deparaffinized in the instrument with EZ prep solution (Ventana Medical Systems). Heat-induced antigen retrieval was carried out using Cell Conditioning 1 (CC1, Ventana Medical Systems) for 32 min at 95 °C. PSMA was detected using clone 3E6 (1/20 dilution, 32 min 37 °C, Agilent/DAKO). Bound antibody was visualized using the OptiView DAB Detection Kit (Ventana Medical Systems). Slides were counterstained with hematoxylin and bluing reagent (Ventana Medical Systems).

## Results

**Fig. 1:**
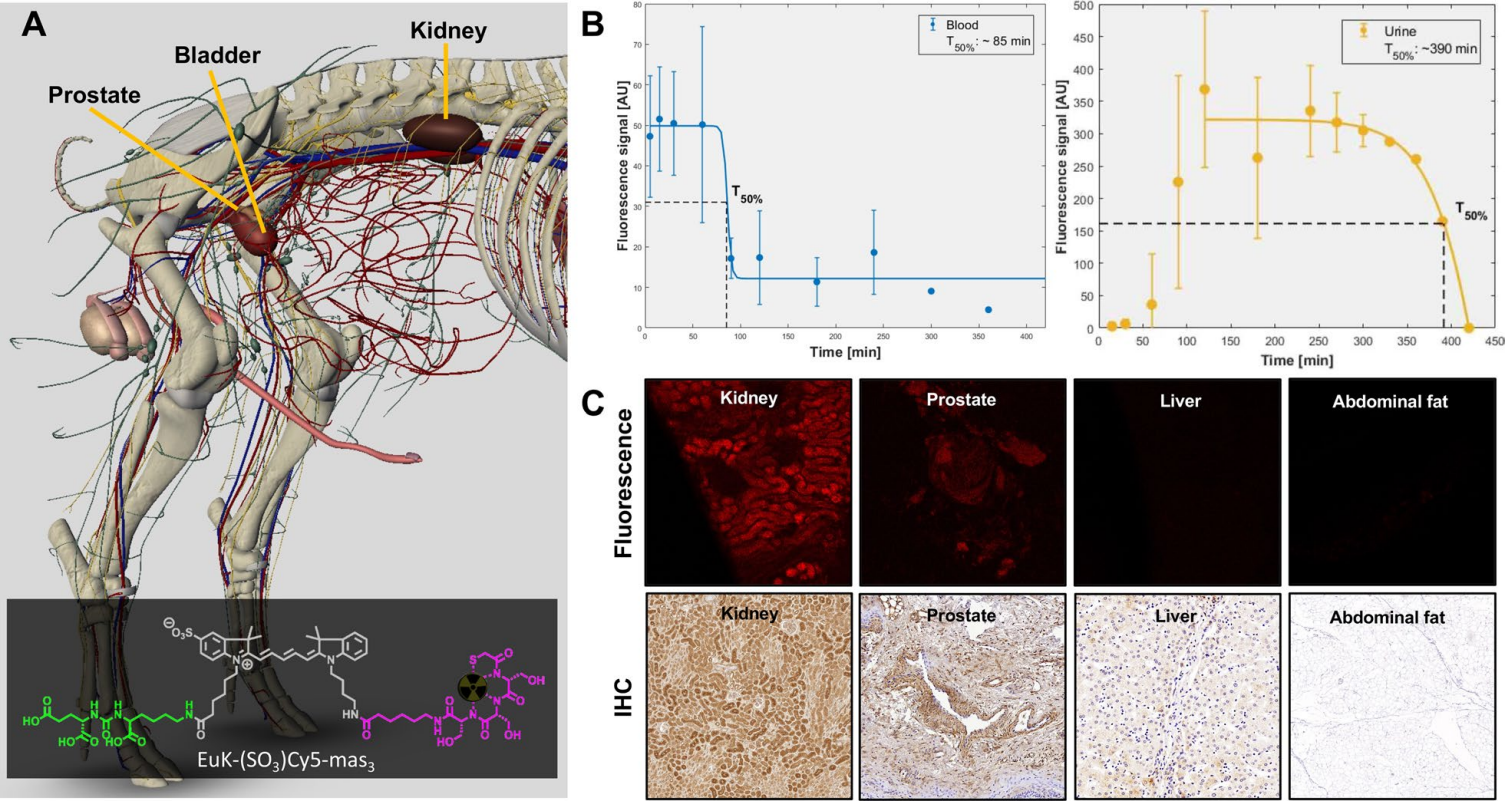
Excretion of EuK‐(SO_3_)Cy5‐mas_3_ in a porcine model. **A** Anatomical overview of vascular and urinary tract (with location of kidney, bladder, and prostate (basal PSMA expression) highlighted). Image was created using 3D-pig-anatomy software from Biosphera. Insert: Structure of EuK‐(SO_3_)Cy5‐mas_3_ [25] with the Euk targeting moiety highlighted in green, the bridging (SO_3_)Cy5 dye in gray and the mas_3_ chelate in pink. **B** Excretion of EuK‐(SO_3_)Cy5‐mas_3_ from the blood (in blue) and urine (in orange) was measured over time after intravenous tracer administration (*T* = 0–*T* = 420 min). **C** Fluorescence confocal microscopy images of fresh excised tissue specimens obtained after in vivo imaging (red on black; top images per organ) and antibody-based PSMA immunohistochemistry (IHC; bottom images per organ) of these tissues

No adverse reactions on breathing and heart rate were observed after administration of 100 µg (2.50–2.86 µg/kg) EuK‐(SO_3_)Cy5‐mas_3_ during the time frame of the experiment (up until 7 h after tracer administration). Conform the %ID/g distribution profile previously reported in mice [25], renal clearance was observed in the pigs (Fig. 1). The fluorescence in urine diminished over time. Assessment of the biological half-life and urine clearance curves (Fig. 1B) indicated that the T_50% blood_ and T_50% urine_ were reached, respectively, at 85 min and 6.5 h (390 min).

**Fig. 2:**
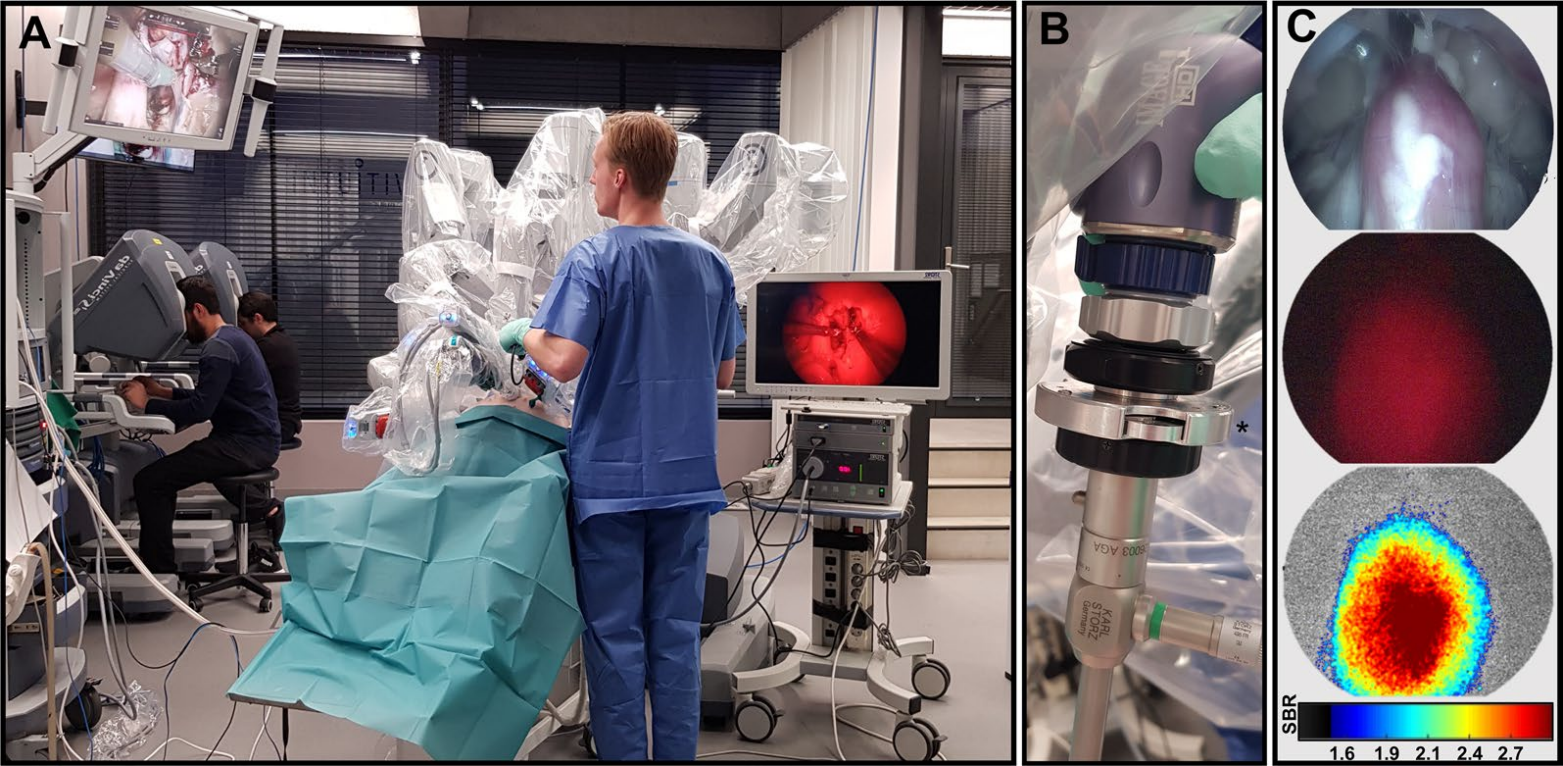
In vivo imaging of basal PSMA levels in the porcine prostate using clinical imaging equipment. **A** Imaging setup showing the operating surgeons (on the left) handing the Da Vinci surgical robot (center) and the Cy5-dedicated laparoscopic imaging setup from KARL STORZ (on the right; [26, 27]). **B**) In-house developed click-on multispectral filter wheel (*) enabling switching between white light imaging and Cy5 imaging. **C** In vivo visualization of the prostate using white light imaging (top), receptor-mediated PSMA imaging (basal expression) after intravenous administration of EuK‐(SO_3_)Cy5‐mas_3_ (center; red on black) and real-time color coding of the fluorescence signal and corresponding signal-to-background ratio (SBR) in the prostate using in-house developed software [26, 27]

During surgery, the Cy5 laparoscope could be effectively inserted through the assistant portal (Fig. 2), a method previously applied in the clinic during fluorescence-guided robotic surgery [31]. White light imaging was used to roughly locate the prostate (Fig. 2C, top image). Placement of the Cy5-specific click-on filter [27] or a filter wheel that facilitated (Fig. 2B, *) easy switching between white light and Cy5 imaging mode (Fig. 2C, red on black image). Visualization of the prostate using fluorescence was shown to be feasible at both early (4 h) and late (7 h) time points after tracer administration (Fig. 2C). Image processing of the fluorescent video-output helped increase the delineation of the tissue that accumulated EuK‐(SO_3_)Cy5‐mas_3_ (Fig. 2C, bottom image). Moreover, the obtained intensity-based rainbow coloring allowed real-time representation of the SBR (Fig. 2C). As fluorescence in urine was considerably lower at 7 h compared to 4 h after tracer administration (Fig. 2B), this would suggest that less contamination due to fluorescent urine would occur at later time points. Unfortunately, in male animals urine was removed directly from the bladder for evaluation of urinary excretion (Fig. 2B), excluding the possibility of evaluating urinary spillage during prostatectomy.

Fluorescence confocal microscopy confirmed EuK‐(SO_3_)Cy5‐mas_3_ uptake in the prostate and also revealed a clear fluorescence signal in the kidneys, but not in the liver or abdominal fat specimens (Fig. 1C). These findings, and the fact that no staining was observed in muscle, splenic tissue, and the salivary gland, are in line with the previously reported biodistribution data of this tracer [25]. In line with literature procedures [28, 29, 32], these results could also be corroborated by PSMA-related IHC (Fig. 1C). Furthermore, no PSMA-specific coloration was seen for the negative control staining.

**Fig. 3:**
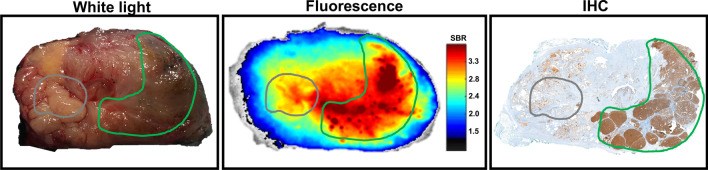
Ex vivo PSMA-mediated imaging prostate cancer in human prostate samples. **A** White light image of prostate tissue obtained after prostatectomy of a prostate cancer patient. **B** Color-coded processing further highlighted the fluorescence uptake. Differences in tracer uptake throughout the tissue after ex vivo tissue incubation with EuK‐(SO_3_)Cy5‐mas_3_ were further highlighted through the representation of signal-to-background ratio (SBR). **C** Corresponding PSMA-related immunohistochemistry of the prostate specimen

In line with earlier evaluations with a different Cy5-tracer [26], a pilot was performed using ex vivo incubation of clinical primary PCa specimens (Fig. 3). Although the approach was subject to tissue deformation between fresh-tissue (white light and fluorescence) and pathologically analyzed fixed tissue (IHC), in all three tissue specimens uptake and fluorescence intensity could be related to the presence of tumor and the expression of PSMA. Again, image processing helped improve the interpretation and helped present the heterogeneity in the fluorescence signal.

## Discussion

Following the intravenous administration of 100 µg EuK‐(SO_3_)Cy5‐mas_3_ [25] the feasibility of surgical visualization of the basal-PSMA expression in the porcine prostate via laparoscopic Cy5-fluorescence imaging could be confirmed (Fig. 2). In addition, porcine studies enabled estimation of the biological half-live and thus the best time point for surgery (Fig. 1). These data could be complemented with proof-of-concept data that indicates EuK‐(SO_3_)Cy5‐mas_3_ can stain human PCa specimens (Fig. 3).

Translation of medical technologies from laboratory to the operating room comes with a number of hurdles that need to be overcome. Two critical ones are toxicity and compatibility with clinical surgical devices.

In clinical trials fluorescent tracers tend to be applied in > 100 µg doses (milligrams per patient; [33, 34]). These high-dose regimes not only significantly increase the cost of the toxicity evaluation but could potentially also lead to saturation of low-capacity targets [35]. Our findings suggest that EuK‐(SO_3_)Cy5‐mas_3_ facilitates fluorescence-guided surgery at a microdosing regimen, despite the fact that the PSMA activity in the pig prostate is eightfold lower compared to the human prostate and PCa yields a tenfold higher PSMA expression level compared to healthy human prostate tissue [28–30, 36]. Although visualization of the prostate was already possible at earlier time points, the presented findings (Fig. 1B) suggest that urine contamination demands an interval between tracer administration and surgery of at least > 7 h. This indicates that a 2-day protocol, similar to what is currently clinically applied for ^99m^Tc-PSMA I&S [6, 10], would be preferred. This also makes the time interval compatible with the current workflow for DROP-IN robotic radioguided surgery [13].

To make sure image-guided technologies match the real-life surgical setting, evaluations were performed in large animal models. For translational prostate cancer research evaluation in dogs or pigs can be envisioned. While dogs can spontaneously develop PCa at an older age, porcine models do not [37]. However, porcine models are regularly used for (robotic) surgical training, providing the opportunity to test the tracer compatibility with robotic surgery and a prototype surgical Cy5 fluorescence endoscope, while adhering to the 3-R principle (replace, reduce, and refine) and reuse animals. A downside of using surgical porcine models is that most facilities designed for surgical training do not permit use of ‘open’ radioactive sources. Hence, the use of radioguidance and single photon emission tomography/computed tomography (SPECT/CT) imaging was prohibited and evaluations of EuK‐(SO_3_)Cy5‐mas_3_ was limited to its fluorescent component. By exploiting the superior brightness of far-red Cy5 fluorescence compared to near-infrared fluorescence [38], our findings further extend the rapidly mounting body of evidence that far-red fluorescence has translational potential. Various clinical studies use Cy5 [26, 27, 39–41] or methylene blue [42, 43]. We refer to our studies in mouse tumor models for the nuclear analysis (SPECT/CT and %ID/g biodistribution data) of the same tracer [25]. Clinical studies with ^99m^Tc-labeled PSMA tracers have shown that the PSMA-targeted radioguidance concept is fully functional in humans below the microdosing limit [10].

Next to the use of large animal models, explanted patient tissue is increasingly being explored to perform proof-of-principle assessment of new imaging strategies and devices in the clinic [26, 27, 44, 45]. Despite only having stained a very small number of clinical specimens, the initial results look promising (Fig. 3) and warrant further investigation of the tracer in ex vivo applications. That said, the true clinical potential of EuK‐(SO_3_)Cy5‐mas_3_ can only be assessed in a clinical study wherein tracer produced under good manufacturing protocols (GMP) is administered intravenously to patients at a microdosing regimen. Further clinical translation of EuK‐(SO_3_)Cy5‐mas_3_ will be aimed at application of both radio and fluorescence guidance during surgery; a formula that has proven to be particularly valuable in robotic PCa surgery using the hybrid tracer ICG-^99m^Tc-nanocolloid [46]. The use of Cy5 for in vivo imaging applications also provides possibilities for exploring multi-color applications in combination with the clinically approved dye ICG [26].

## Conclusions

In a porcine model, intraoperative PSMA-mediated fluorescence imaging was shown to be feasible following intravenous injection of EuK‐(SO_3_)Cy5‐mas_3_ in a microdosing regimen. Proof-of-concept data obtained following ex vivo incubation of human PCa specimen underlined the tracers compatibility with human tissue.

## Data Availability

The datasets used and/or analyzed during the current study are available from the corresponding author on reasonable request.

## Abbreviations

PSMA: Prostate-specific membrane antigen
PCa: Prostate cancer
RP: Radical prostatectomy
Cy5: Cyanine-5
ICG: IndoCyanine green
SBR: Signal-to-background ratio
RARP: Robot-assisted radical prostatectomy
NKI-AvL: Netherlands Cancer Institute-Antoni van Leeuwenhoek Hospital
TBS: Tris-buffered saline
TBST: Tris-buffered saline and Tween 20
IHC: Immunohistochemistry
SPECT/CT: Single photon emission computed tomography/computed tomography
